# Maternal death of a hemophilic patient due to the inhaling of a mixture of industrial bleach and detergents—A case study

**DOI:** 10.1002/ccr3.3535

**Published:** 2020-11-12

**Authors:** Parvin Abedi, Zaynab Mohaghegh, Nahid Faramazi, Zahra Biygom Seyyed Aghamiri

**Affiliations:** ^1^ Department of Midwifery Menopause Andropause Research Center Ahvaz Jundishapur University of Medical Sciences Ahvaz Iran; ^2^ Department of Family Health Health Deputy of Tehran University of Medical Science Tehran Iran; ^3^ Department of Family Health Health Deputy of Yasuj University of Medical Sciences Yasuj Iran

**Keywords:** bleach, detergents, maternal mortality, poisoning

## Abstract

Use of detergents and cleaning products puts women at risk due to hazardous chemical substances. Education of all women, especially pregnant and high‐risk women about the proper use of detergents, is a necessity.

## Graphical abstract

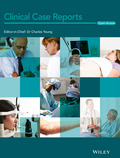

## 1 INTRODUCTION

Today, bleach and detergents are the most commonly used products in the society. People may use a mixture of detergent and bleach to achieve extracleaning, a combination which, due to chemical reactions, releases large amounts of chlorine, resulting in poisoning symptoms when inhaled. In this case report, we discuss a maternal death of a hemophilic pregnant woman after using a combination of bleach and detergents, in her early stage of pregnancy.

Poisoning is one of the major public health problems and is one of the most common reasons for medical emergencies whether caused intentionally or accidentally.^1^ According to the American Association of Poison Control in 2017, the total cases of toxic poisoning during pregnancy were 0.321% of all human exposures. The rate of poisoning in the first, second, and third trimesters of pregnancy was reported 37.1%, 34.9%, and 28.0%, respectively. Most of these cases are unintentional. Moreover, two deaths from poisoning during pregnancy were also reported.^2^

In many studies, household chemicals such as detergents, bleach, and oil have been mentioned as the most common causes of poisoning.^3^, ^4^, ^5^, ^6^ National Poison Data System of America (2017) revealed that the poisoning rate with household detergents is to be 7.43%.^2^

Detergents are substances used to clean grease and dirt particles from wipe cloths, surfaces, or other objects and are prepared in various forms. Bleach and detergents are among the most commonly used products.^7^ Bleaches are chemicals having disinfectant and bleaching properties acting through a process of oxidation. Household or chlorine bleach contains hypochlorite (NaCLO), also known as Whitex. This water‐diluted bleach comes at 5% or 10%. Whitex is an unstable solution, strong oxidizer, and alkaline substance that because of high pH (Ph ~ 11) is so corrosive..^8^

People may use a mixture of detergent and bleach to achieve extracleaning, a combination which releases large amounts of chlorine, resulting in poisoning symptoms when inhaled.^9^ Improper use of these compounds, especially in confined areas, irritates the airways because of chlorine and symptoms of poisoning occur in people.^10^, ^11^ The chlorine is a colorless to yellow‐green color gas with a unique and irritating odor. This element has become intertwined with human life.^12^, ^13^ However, in 1915 it was used as a chemical gas during World War I.^14^, ^15^

Chlorine gas may use as one of the constituents of bleach ^8^ Besides, it should be noted that the mixing of bleach and detergent releases a large amount of chlorine gas, causing strong adverse effects.^9^, ^16^ Studies have shown that the inhalation is the main route of exposure to chlorine.^6^ Chlorine gas is heavier than air and is an oxidizing agent that reacts with water in the body tissues, releasing hypochlorous acid, hypochloric acid, and free oxygen radicals, which are considered tissue toxins.^17^

The most important adverse effects of chlorine in the body are caused by the creation of free radicals of oxygen. However, oxygen, is an element that sustains life, can have strong harmful effects on the body under certain circumstances.^18^ Chlorine is a respiratory system stimulant that can affect every part of the body with a high concentration of water. The main organs affected by chlorine gas include eyes, mucous of the nose membranes, pharynx, larynx, trachea, and bronchi.^17^, ^19^

## 2 PATIENT INTRODUCTION

A 31‐year‐old nulliparous woman with a history of hemophilia, and gestational age of 20 weeks, was referred to the emergency department complaining of lethargy and cough. An initial examination showed that the patient had exposure to mixed hydrochloric acid with Whitex in warm water when cleaning the floor three hours ago. She was treated in an emergency department as an outpatient. The woman was normal after discharge until the night. Then, she was referred back to the emergency department at 5 AM with symptoms of lethargy, severe dyspnea, cyanosis, and hematemesis, and was hospitalized with a diagnosis of upper gastrointestinal tract bleeding.

At 6:45 AM, she was transferred to the intensive care unit, and the reservation of 40 P.C and oxygen flow was established for her. Vital signs were as follows: BP = 120/70 mmHg, GCS score = 13/15, PR = 120, RR = 20, and T = 37°C. The patient was complaining of cough and hemoptysis. The fetal heart was normal. Dextromethorphan syrup and acetaminophen codeine pill were prescribed.

The patient was extremely agitated and restless. The heart monitoring showed sinus rhythm and tachycardia. Six units of the FFP were reserved, and a pack of Factor 8 was transfused. At 12:15 PM, becotide and salbutamol spray were prescribed and the authorities decided to transfer the patient to a more equipped hospital. Admission permission to a level 3 hospital was issued, and the patient was transferred.

She was admitted to the ICU in a referral hospital with BP = 150/70 mmHg, PR = 140, RR = 40, T = 37°C, and emphysema around the neck area, pulmonary hemorrhage, and dyspnea and was connected to the ventilator. Despite the prescription of fentanyl and midazolam, the patient was highly uncomfortable under the machine.

On the third day of hospitalization, the GCS score of patient was 6/15. The patient received cryotherapy and Factor 8. The amount of urine was 600 mL/24h with hematuria. Propofol (14 drops/min) was administered, and because of the patient's tachypnea, the dose was increased to improve the respiratory condition. The patient had bloody discharge from her mouth.

Four days after admission, the patient's vital signs were as follows: Platelets = 50,000/uL, RR = 18, PR = 120, BP = 107/54 mmHg, T = 39°C, urine output = 1000 cc per 24h, and bruises throughout the patient's body. Pulmonary emphysema spread to the lower regions. Chest X‐ray did not show any symptoms of pneumothorax.

At the end of the fifth day of hospitalization, the platelets count was 15,000/uL, and white blood cell count was 30,000/uL. Around 3 AM of 6th day of admission, blood pressure dropped again, and due to tachycardia, low‐dose dopamine was prescribed, but no appropriate response was observed. Following the treatment, because of vomiting with blood clots, the lavage was given. Pulmonary hemorrhage and cardiac arrest happened around 6 AM CPR was started and lasted about an hour and a half but was not successful, and the patient was expired at 7:30. Pulmonary hemorrhage caused by the inhalation of toxic gases was reported as the cause of death.

## 3 DISCUSSION

Bleach refers to a large class of compounds used to whitening or discolored materials. They are often used for cleaning and disinfection. Bleachers kill or control most viruses, bacteria, molds, and algae. There are industrial and concentrated forms of bleach that are used to clean equipment and water treatment.^20^ Bleach is one of the most widely used household detergent that used for surface cleaning and disinfection, cleaning clothes, and treating fabric. Improper use, especially when mixed with other detergent, can be harmful or even fatal.^21^ If bleach mix with other household cleaners such as toilet bowl cleaners and ammonia, it can be very dangerous.^22^

Most women do not have enough knowledge about the safe and proper storage and use of detergents. The use of a mixture of detergent and bleach, especially in environments without proper ventilation, affects the body because of releasing chlorine gas. Inhalation of this gas has detrimental effects such as lung irritation, damage to the throat, headache, and shortness of breath. Respiratory tract irritation is the first sign of detergent entry into the respiratory system followed by coughing, and hematological complications may cause death in long term ^4^, ^18^ These symptoms are more evident among those who had mixed several detergents.^23^

The results of the studies showed that inhaling of a mixture of bleach and detergents results in increased leukocytes in mice.^24^ Inhalation of chlorine and its reaction with water in body tissues cause the release of oxygen‐free radicals, which are extremely unstable molecules. The oxygen‐free radicals tend to take electrons from other molecules, transforming the victim's molecules into free radicals, thus establishing chain reaction in the production of free radicals.^25^, ^26^ Free radicals have destructive effects at four points of the body including lipid compounds, proteins, DNA, and lysosomes. As a result, cells lose their ability to get nutrients and have cellular exchanges, leading to cell death.^25^ This process of damages to respiratory system tissues stimulates the immune system, and, in many cases, this can lead to acute lung injury.^3^ The studies showed that around 6.3% of women in Iran used a mixture of several detergents for achieving extra cleaning effects.^23^ In Canada, every year, one million cases of poisoning occur caused by using household cleaning products, and many of them are fatal. The frequency of inhaling household detergent in India has reported being 7% between 1999 and 2002.^27^

Scientific findings have shown that the concentration of chemical poisoning caused by detergents indoor is five times more than outdoor.^28^ Pregnant women are at increased risk, especially if they have hemophilia.^29^

Hemophilia, which in turn is a risk factor for bleeding during pregnancy, along with other underlying conditions such as congenital disorders of blood coagulation, is one of the rare causes of death among pregnant women and threatens the life of mother and fetus.^30^ Kadir et al (1997) reported that 20% of women with hemophilia had a postpartum hemorrhage and, two of them, had severe bleeding.^31^ Hemophilia A and B are sex‐linked bleeding disorders created respectively by defects in the genes of Factor 8 and Factor 9. The “obstetric” bleeding risk in each of these hemophilia cases is directly affected by the Factor VIII or Factor IX.^31^

A patient's condition with hemophilia during pregnancy can be clinically and experimentally normal without any history of bleeding during pregnancy. However, lack of awareness about the correct use of detergents by patients has created a fertile ground for blood coagulation disorders. Clinical and blood disorders such as emphysema in the neck and chest, thrombocytopenia, increased white blood cell count, renal failure, and other symptoms indicate the severity of damage to the respiratory system. Any delay in treatment can result in irreparable results.

## 4 CONCLUSIONS

The indoor use of detergents and cleaning products puts women at risk due to hazardous chemical substances. Education of all women, especially pregnant and high‐risk women about the proper maintenance and use of detergents, is a necessity. It is suggested that in cases of poisoning of pregnant women they should be monitored for at least 24 hours.

## CONFLICT OF INTEREST

None declared.

## AUTHOR CONTRIBUTIONS

PA: is a primary manuscript author; ZA: involved in editing the manuscript; NF: followed the patient; ZM: is an overall senior author and followed the patient.

## ETHICAL APPROVAL

This manuscript does not contain any studies on human participants or animals.

## Data Availability

Data sharing not applicable to this article as no datasets were generated or analyzed during the current study.

## References

[1] Lamireau T , Llanas B , Kennedy A , et al. Epidemiology of poisoning in children: a 7‐year survey in a paediatric emergency care unit. Eur J Emerg Med. 2002;9(1):9‐14. 11989508 10.1097/00063110-200203000-00004

[2] Gummin DD , Mowry JB , Spyker DA , Brooks DE , Osterthaler KM , Banner W . 2017 Annual Report of the American Association of Poison Control Centers' National Poison Data System (NPDS): 35th Annual Report. Clin Toxicol (Phila). 2018;56(12):1213‐1415. 30576252 10.1080/15563650.2018.1533727

[3] Gupta SK , Peshin SS , Srivastava A , Kaleekal T . A Study of Childhood Poisoning at National Poisons Information Centre, All India Institute of Medical Sciences, New Delhi. J Occup Health. 2003;45(3):191‐196. 14646297 10.1539/joh.45.191

[4] Hoffman RJ , Osterhoudt KC . Evaluation and management of pediatric poisonings. Pediatr Case Rev. 2002;2(1):51. 12865696 10.1097/00132584-200201000-00007

[5] Motlagh M , Nazari Z .Epidemiologic study of pediatric poisoning in amir kabir and abozar hospital of ahvaz in the year 2000. 2002.

[6] Yates KM . Accidental poisoning in New Zealand. Emerg Med (Fremantle, WA). 2003;15(3):244‐249. 10.1046/j.1442-2026.2003.00443.x 12786646

[7] Grime K , Clauss A . Laundry bleaches and activators. Chemist Ind. 1990;647‐649.

[8] Dence CW , Reeve DW . Pulp bleaching. Principles and Practice TAPPI Atlanta USA. 1996.

[9] Trotman ER .Textile scouring and bleaching 1968.

[10] Das R , Blanc PD . Chlorine gas exposure and the lung: a review. Toxicol Ind Health. 1993;9(3):439‐455. 8367885 10.1177/074823379300900304

[11] Jiang X , Buckley L , Morgan K . Pathology of toxic responses to the RD50 concentration of chlorine gas in the nasal passages of rats and mice. Toxicol Appl Pharmacol. 1983;71(2):225‐236. 6636187 10.1016/0041-008x(83)90339-3

[12] Gilchrist HL , Matz PB . The residual effects of warfare gases: the use of chlorine gas, with report of cases. Med Bull Vet Admin. 1933;6:229‐270.

[13] Schwartz D . Acute inhalational injury. Occup Med. 1987;2(2):297‐318. 3303382

[14] Klonne D , Ulrich C , Riley M , Hamm T Jr , Morgan K , Barrow C . One‐year inhalation toxicity study of chlorine in rhesus monkeys (Macaca mulatta). Fundam Appl Toxicol. 1987;9(3):557‐572. 3692014 10.1016/0272-0590(87)90037-6

[15] Martinez TT , Long C . Explosion risk from swimming pool chlorinators and review of chlorine toxicity. J Toxicol Clin Toxicol. 1995;33(4):349‐354. 7629902 10.3109/15563659509028921

[16] Gapany‐Gapanavičius M , Yellin A , Almog S , Tirosh M . Pneumomediastinum: a complication of chlorine exposure from mixing household cleaning agents. JAMA. 1982;248(3):349‐350. 7087130 10.1001/jama.248.3.349

[17] Barrow CS , Alarie Y , Warrick JC , Stock MAF . Comparison of the sensory irritation response in mice to chlorine and hydrogen chloride. Arch Environ Health. 1977;32(2):68‐76. 849012 10.1080/00039896.1977.10667258

[18] Bagchi K , Puri S . Free radicals and antioxidants in health and disease: A review. 1998.

[19] Jones RN , Hughes JM , Glindmeyer H , Weill H . Lung function after acute chlorine exposure. Am Rev Respir Dis. 1986;134(5):1190‐1195. 3789518 10.1164/arrd.1986.134.6.1190

[20] Kucuk G , Gollu G , Ates U , et al. Evaluation of esophageal injuries secondary to ingestion of unlabeled corrosive substances: pediatric case series. Arch Argent Pediatr. 2017;115(2):e85‐e88. 28318189 10.5546/aap.2017.eng.e85

[21] Arif AA , Delclos GL . Association between cleaning‐related chemicals and work‐related asthma and asthma symptoms among healthcare professionals. Occup Environ Med. 2012;69(1):35‐40. 21602538 10.1136/oem.2011.064865

[22] Benzoni THJ . Bleach Toxicity. Island: StatPearls Publishing; Updated 2020 Jun 29. 28722950

[23] Atta E , Gardner M . Cardiopulmonary resuscitation in pregnancy. Obstet Gynecol Clin North Am. 2007;34(3):585‐597. 17921016 10.1016/j.ogc.2007.06.008

[24] Vaezi G , Pourkazem M , Toosi F , Aliabadi F . Effects Aerosol of Industrial Bleach and Detergent Mixture on Mucosa Layer and Lamina Mucosa Conjunctiva in Mice. J Chem Health Risks. 2018;3(1):1–5.

[25] Derick H , Williams E , Cadenas E . Mitochondrial respiratory chain‐dependent generation of superoxide anion and its release into the intermembrane space. Biochemical J. 2001;353(2):411‐416. 10.1042/0264-6021:3530411 PMC1221585 11139407

[26] Patel RP , Cornwell T , Darley‐Usmar VM . The biochemistry of nitric oxide and peroxynitrite: implications. understanding the process of aging: the roles of mitochondria. Free Radicals, and Antioxidants. 1999;39.

[27] Srivastava A , Peshin SS , Kaleekal T , Gupta SK . An epidemiological study of poisoning cases reported to the National Poisons Information Centre, All India Institute of Medical Sciences, New Delhi. Hum Exp Toxicol. 2005;24(6):279‐285. 16004194 10.1191/0960327105ht527oa

[28] Shapiro JM . Critical care of the obstetric patient. J Intensive Care Med. 2006;21(5):278‐286. 16946443 10.1177/0885066606290390

[29] Mansouri A , Hejazi A , Badieian MN . A survey on mortality among pregnant women and its causes in cases referred to Khorasan Legal Medicine center between 1999–2005. 2005.

[30] Pritchard JA , MacDonald PC , Gant NF .Williams obstetrics: Appleton‐Century‐Crofts; New York; 2010.

[31] Kadir R , Economides D , Braithwaite J , Goldman E , Lee C . The obstetric experience of carriers of haemophilia. BJOG. 1997;104(7):803‐810. 10.1111/j.1471-0528.1997.tb12024.x 9236645

